# Compost Addition Enhanced Hyphal Growth and Sporulation of Arbuscular Mycorrhizal Fungi without Affecting Their Community Composition in the Soil

**DOI:** 10.3389/fmicb.2018.00169

**Published:** 2018-02-07

**Authors:** Wei Yang, Siyu Gu, Ying Xin, Ayodeji Bello, Wenpeng Sun, Xiuhong Xu

**Affiliations:** ^1^College of Resources and Environment, Northeast Agricultural University, Harbin, China; ^2^Institute of New Rural Development, Northeast Agricultural University, Harbin, China

**Keywords:** arbuscular mycorrhizal, compost addition, growth stage, black soil, soybean

## Abstract

Arbuscular mycorrhizal (AM) fungi form symbiotic associations with most crop plant species in agricultural ecosystems, and are conspicuously influenced by various agricultural practices. To understand the impact of compost addition on AM fungi, we examined effect of four compost rates (0, 11.25, 22.5, and 45 Mg/ha) on the abundance and community composition of AM fungi in seedling, flowering, and mature stage of soybean in a 1-year compost addition experiment system in Northeast China. Soybean [*Glycine max* (L.) Merrill] was used as test plant. Moderate (22.5 Mg/ha) and high (45 Mg/ha) levels of compost addition significantly increased AM root colonization and extraradical hyphal (ERH) density compared with control, whereas low (11.5 Mg/ha) level of compost addition did not cause significant increase in AM root colonization and ERH density. AM fungal spore density was significantly enhanced by all the compost rates compared with control. The temporal variations analysis revealed that, AM root colonization in seedling stage was significantly lower than in flowering and mature stage. Although AM fungal operational taxonomic unit richness and community composition was unaffected by compost addition, some abundant AM fungal species showed significantly different response to compost addition. In mature stage, *Rhizophagus fasciculatum* showed increasing trend along with compost addition gradient, whereas the opposite was observed with *Paraglomus* sp. In addition, AM fungal community composition exhibited significant temporal variation during growing season. Further analysis indicated that the temporal variation in AM fungal community only occurred in control treatment, but not in low, moderate, and high level of compost addition treatments. Our findings highlighted the significant effects of compost addition on AM growth and sporulation, and emphasized that growth stage is a stronger determinant than 1-year compost addition in shaping AM fungal community in black soil of Northeast China.

## Introduction

Agricultural development in countries like China is generating substantial amounts of organic waste. By the year 2010, the annual wastes of straw and manure generated were up to 0.8 and 2.12 billion ton, respectively (Bi et al., 2010; Zhu and Ma, 2014). These large amounts of wastes are under-utilized and thereby causing a serious threat to the environment. Composting of organic solid wastes is an effective strategy for organic waste recycling (Santos et al., 2011) and beneficial practice for soil restoration (Scotti et al., 2016). Compost application not only provided an important source of nutrients (Duong et al., 2012), but also improved soil structure (Celik et al., 2004), enhanced crop yield, and suppressed soil-borne pathogens (Pane et al., 2013). Compost amendments therefore maintain and enhance the fertility and productivity of agricultural soils (Pérez-Piqueres et al., 2006).

Arbuscular mycorrhizae (AM) are symbiotic associations formed between terrestrial plant roots and soil fungi of the Glomeromycota (Smith and Read, 2008). The large majority of agricultural crops, such as wheat, rice, corn, potato, and soybean form symbiosis with AM fungi (Hijri, 2016). In the AM association, AM act as an extension of root systems and increase the surface area that is used for nutrient absorption (Smith and Read, 2008). AM fungi benefit their host principally by increasing uptake of phosphorus (Marx, 2004). Moreover, AM fungi can transfer both inorganic and organic N to the host plant from soil (Hodge et al., 2001; Barrett et al., 2011; Saia et al., 2014b; Hodge and Storer, 2015). Beside macronutrients absorption, AM fungi also enhance plant growth by uptaking of some micronutrients, enhancing tolerance to soil pathogens and abiotic stresses (Smith and Read, 2008). AM fungi, due to their key role in plant growth, could improve crop yield and quality (Hijri, 2016). In this view, AM fungi gained a growing interest as ecosystem engineers and biofertilizers (Igiehon and Babalola, 2017) and are considered one of the most important soil microorganisms for agroecosystem.

Fertilization is an important agricultural practice to increase crop yields (Liu et al., 2016). AM fungi, as key interface between plant hosts and soil mineral nutrients, have been demonstrated to respond to a variety of inorganic and organic fertilizers (Oehl et al., 2004; Liu et al., 2012; Cavagnaro, 2014). It was well demonstrated that use of inorganic fertilizer (e.g., N and P fertilizer) negatively affect AM abundance and diversity (Liu et al., 2012; Watts-Williams and Cavagnaro, 2012). Such effects can be due to the excessive soil N and P, to the changes in soil pH, and to the indirect effect of fertilization on plant community and productivity (Liu et al., 2012). On the contrary, AM fungal growth was generally enhanced by organic fertilization (Oehl et al., 2004; Alguacil et al., 2011; Zhu et al., 2016). Compost is increasingly used as an organic fertilizer (Cavagnaro, 2014), it can slowly release nutrients for plants and microbes and help maintaining a medium-high nutrient availability (Scotti et al., 2016; Yang et al., 2017), which may benefit AM fungi. Although AM fungi are not saprotrophic fungi, some studies have shown that AM fungi can directly take advantage of organic matter (Hodge et al., 2001; Govindarajulu et al., 2005; Jin et al., 2005). In addition, compost addition usually promotes plant growth and enhances carbon allocation to soil fungi (Lee et al., 2004; Donn et al., 2014), thus can indirectly affect AM fungi. The effect of compost addition on AM can also be mediated through soil bacteria, which was reported to either directly enhance AM growth and germination or indirectly by influencing plant physiology (Saia et al., 2015a,b). Taken as a whole, the addition of compost mostly had beneficial effect on AM growth. A number of studies have reported that compost addition enhanced AM root colonization, spore production, and development of AM extraradical hyphae (Labidi et al., 2007; Valarini et al., 2009; Tanwar et al., 2013; Cavagnaro, 2015). However, negative or neutral effect of compost addition on AM biomass was sometimes reported in field or greenhouse studies (Copetta et al., 2011; Cozzolino et al., 2016). In a review paper, Cavagnaro (2015) found that 8% publications detected a negative effect of compost addition on AM root colonization. In addition, compost showed positive or negative effect on AM fungal extraradical hyphal (ERH) density, depending on the plant species (Valarini et al., 2009). The discrepant effects indicated that the effect of compost addition on AM fungi should still be confirmed through further investigations. Moreover, understanding the diversity and community composition of AM fungi is prerequisite for the understanding of its ecological function (Njeru et al., 2015). Previous research showed that the application of manure or other organic fertilizers usually had positive effects on AM fungal diversity and modified the AM fungal community composition (Oehl et al., 2004). However, little information is available on the effect of compost addition on AM fungal community composition as far as we know.

On the other hand, growth stage of host plant has been suggested as another important factor influencing AM fungal hyphal growth and soil AM community composition as AM growth is dependent on the carbon provided by host plant (Dumbrell et al., 2011; Bainard et al., 2012). Several studies indicated that plant growth stage exerted stronger influence in AM fungal biomass, soil AM fungal diversity and community composition than agricultural practices (Tian et al., 2011; Wu et al., 2011; Njeru et al., 2015). For instance, AM fungal spore community and biomass were mainly affected by growth stage rather than agroecosystem management in an intensively managed maize agroecosystem in North China (Tian et al., 2011). AM root colonization exhibited strong temporal variation in a tallgrass prairie ecosystem but minimally affected by nitrogen enrichment (Mandyam and Jumpponen, 2008). Although temporal variations of AM was well studied in various ecosystems (Dumbrell et al., 2011; Bainard et al., 2012; Barnes et al., 2016), temporal variations of AM fungi under compost addition condition is unknown.

Black soil is a vital soil resource for crop production distributed in Northeast China (Xu et al., 2010; Yin et al., 2015). However, serious soil erosion and fertility deterioration has occurred in this region over the past several decades (Liu and Diamond, 2005; Yao et al., 2017). A controlled gradient compost addition experimental system has therefore been established in an agroecosystem on the Songnen Plain to study the responses of plants, soil fertilities and microorganisms to compost addition. The aim of the present study was to (1) determine whether the AM root colonization, spore density, ERH density, and diversity are enhanced by compost addition; (2) whether there is dose-dependent effect of compost addition on AM fungal biomass, diversity, and community composition; and (3) whether there is temporal variation in the AM fungal growth and community under compost addition condition.

## Materials and Methods

### Field Experiment Design

A field experiment was conducted at the experimental farm station, Northeast Agriculture University, eastern Songnen Plain, China (45°45′45″ N, 126°54′46″ E) in 2016. This region has a typical monsoon climate, with annual average temperature of about 4–5.5°C, and annual precipitation about 400–500 mm (70–80% during summer, Ding et al., 2017). The soil is classified as phaeozem (according to World Reference Base for Soil Resources), with pH of 6.3, soil organic matter (SOM) of 25.8 g/kg, total N of 1.1 g/kg, total P of 0.5 g/kg. The field was ploughed to the depth of 30 cm before planting and seed were sown in rows and with a density of 30 plants m^-2^.

Soybean [*Glycine max* (L.) Merrill], Dongnong, 53 genotype was planted on May 6 and harvested on September 29, 2016. The compost used was obtained through 45 days on-farm composting of cow manure and maize straw. Chemical analyses of compost showed a pH of 8.0, 386.1 g/kg total organic carbon, 18.4 g/kg total N, 1008.1 mg/kg available P, 399.8 mg/kg NO_3_-N, 212.9 mg/kg NH_4_-N, and C:N ratio of 21.0. Four levels of compost were applied as basal fertilizer before soybean was planted: (1) no compost addition (NC); (2) 11.25 Mg/ha compost addition (low level of compost addition, LC); (3) 22.5 Mg/ha compost addition (moderate level of compost addition, MC); and (4) 45 Mg/ha compost addition (high level of compost addition, HC). The compost application rate in LC treatment was approximately equal to 200 kg N/km^2^ (the recommended amount of N fertilizer in this area). The treatments were arranged in a randomized block design with four replicates (16 plots in total, plot size 5 m × 4.5 m each and 2 m separation from each other). Weeds were removed manually twice during the growing season, no other fertilizers, herbicide, or rhizobium inoculant were added.

### Sampling

Soil and root samples were collected in seedling, flowering, and mature stage, respectively. Sampling times were as follows: June 4 (seedling stage), July 24 (flowering stage), and August 27, 2016 (mature stage). Briefly, five soil cores (near plant roots, 20 cm deep, 5 cm diameter) and soybean roots from each plot were randomly collected and mixed as one composite sample. In total, 48 soil and root samples for the 3 sampling times were collected, then packed in an ice box and transported to laboratory. Fresh soil samples were sieved (1-mm sieve) to remove roots and debris. Soybean roots (< 1 mm diameter) were washed with sterilized deionized water and dried up with filter paper. Fresh soil and root samples were then stored at -80°C for further analyses.

### Soil Physical and Chemical Analyses

Soil variables were determined by Yang et al. (2017) after all soil sampling. Briefly, soil pH was determined in 1:2.5 (v/v) soil/water extracts using a combination glass electrode. Soil gravimetric moisture was determined by drying at 105°C for 48 h. Soil bulk density was determined from the oven-dried undisturbed cores as the mass of the dried soil per volume. SOM content was determined by the potassium dichromate oxidation-ferrous sulfate titrimetric (Walkley and Black, 1934). Total N (TN) was determined as ammonium-N by steam distillation after digestion with H_2_SO_4_ (Bremner and Mulvaney, 1982). Available N (AN) was determined by the alkaline hydrolysis diffusion method. Total P (TP) was measured by digesting soil using the H_2_SO_4_–HClO_4_ method and then measured using Mo–Sb colorimetry method (Murphy and Riley, 1962); available P (AP) was extracted using 0.5 mol/L NaHCO_3_ solution and determined as described above. The available K (AK) was extracted with NH_4_OAc and determined by flame atomic absorption spectrometry (Schollenberger and Simon, 1945). Soil microbial biomass carbon (MBC) was measured using the chloroform fumigation extraction method (Vance et al., 1987). Biolog EcoPlate^TM^ (BIOLOG Inc., Hayward, CA, United States) was used to characterize soil microbial community.

### AM Root Colonization, ERH Density, and Spore Density

A total of 50 fine root fragments (ca. 1 cm long) of each sample were stained with acid fuchsin and the percentage of AM root colonization was quantified by the magnified line-intersect method (McGonigle et al., 1990). Extraction of AM fungal ERH was followed by Rillig et al. (1999). Hyphae were distinguished into mycorrhizal and non-mycorrhizal hyphae based on their morphology and staining color according to Miller et al. (1995) at 200 × magnification, and the hyphal length was measured by a line intersection method. AM fungal spores were extracted from 20.0 g air-dried soil of each sample with deionized water using the wet-sieving and decanting method and counted under 50 × magnification (Daniels and Skipper, 1982).

### Molecular Analysis of AM Fungi

Genomic DNA was extracted from 0.25 g fresh soils with a PowerSoil DNA Isolation Kit (Mo Bio Laboratories, Carlsbad, CA, United States) following the manufacturers’ instruction. An approximately 334 bp region of the 18S rDNA gene was amplified with two-step PCR. The first amplification with primers GeoA-2 (Schwarzott and Schüßler, 2001) and AML2 (Lee et al., 2008) was carried out in a final 25 μL reaction solution including 2.5 μL 10 × buffer, 1.5 mM MgCl_2_, 200 μM of each dNTP, 0.75 μM of each primer, 0.75 U PrimeSTAR HS DNA Polymerase (Takara, Japan), and 1 μL template DNA. The thermal cycling was followed by an initial denaturation at 94°C for 5 min, 30 cycles of denaturation at 94°C for 45 s, annealing at 56°C for 1 min, and extension at 72°C for 1.5 min, followed by a final extension at 72°C for 10 min. The products of the first amplification were diluted for 10 times with sterilized deionized water and 1.0 μL diluted solution was used as the template for the nested PCR. Primers AMDGR (Lumini et al., 2010) and NS31 (Simon et al., 1992) were used for the nested PCR, and the forward primer was modified with a unique 12 nt barcode at the 5′ end. Conditions for the nested PCR were similar to the first PCR, except for 58°C annealing temperature and extension for 1 min. PCR products were purified using an agarose gel DNA purification kit (TaKaRa, Japan) and quantified using Nanodrop 2000 (Thermoscientific, United States). Only PCR products with concentration > 10 ng/μL and OD 260/OD 280≈1.8 were used, others were discarded and re-amplified to ensure the MiSeq sequencing quality. The final PCR products from all samples were mixed at equimolar concentrations and then subjected to Illumina MiSeq platform at Environmental Genome Platform of Chengdu Institute of Biology, Chinese Academy of Sciences. The raw sequence data had been accessioned in the Sequence Read Archive of National Center for Biotechnology Information, United States (accession numbers: SRR6431651–6431666, SRR6431681–6431696, SRR6431703–6431718).

### Bioinformatics Analysis

Raw sequences with low quality (length < 250 bp, with ambiguous base ‘N,’ and average base quality score < 20) were removed using QIIME Pipeline Version 1.8.0 (Caporaso et al., 2010) before further analysis. Potential chimeras were discarded using the ‘chimera.uchime’ command in Mothur (Schloss et al., 2009), using both no external database and the MaarjAM 18S rRNA gene reference database (Öpik et al., 2010). The remaining non-chimeric sequences were clustered into different operational taxonomic units (OTUs) with 97% similarity level using USEARCH v8.0 (Edgar, 2013) after dereplication and discarding all singletons. The representative sequences (the most abundant one for each OTU) from OTUs were blasted against the NCBI nt database (Altschul et al., 1990), and all non-AM fungal OTUs were removed from dataset (identified based on closest BLAST hit not annotated as ‘Glomeromycota’). Then the AM fungal OTUs were confirmed by a ‘blastn’ search in the MaarjAM 18S rRNA gene database using an *E*-value less than 1e^-50^ as a significant matching criterion (Öpik et al., 2010). To account for the variation in read numbers among samples, the number of sequences per sample was normalized to the smallest sample size using the ‘normalized.shared’ command in the Mothur (Schloss et al., 2009). We then constructed a neighbor joining tree including certain taxa of Glomeromycota from GenBank in MEGA v5 (Tamura et al., 2011) to identify AM fungal OTUs. Accumulative numbers of AM fungal OTUs were calculated using the ‘rarefy’ function in the package Vegan (Oksanen et al., 2013) in R (R Core Team, 2013). The representative sequences of the AM fungal OTUs at the 97% nucleotide identity were submitted to the International Nucleotide Sequence Database Collaboration (accession numbers: MF567529–MF567573).

### Statistical Analysis

Two-way ANOVAs were used to examine the effects of compost addition, growth stage, and their interaction on AM root colonization, spore density, ERH density, OTU richness, Shannon diversity indices, and net-relatedness indices (NRIs). All data met the assumptions of sphericity (*P* > 0.05 in Mauchly’s test of sphericity). All data were tested for normality and homogeneity of variance before two-way ANOVA. Differences among compost addition treatments and plant growth stages were tested using the one-way ANOVA followed by a Tukey’s HSD *post hoc* test at *P* < 0.05.

Structural equation model (SEM) was used to detect the direct and indirect effect of compost addition on AM root colonization, spore density, and ERH density using AMOS (Arbuckle, 2011). We assumed *a priori* model based on our knowledge of soil ecological causal relationships. Soil AP was log-transformed in order to achieve normal distribution. Model adequacy was determined by χ^2^ tests (*P* > 0.05), goodness-of-fit index (GFI > 0.9), Akaike Information Criteria (AIC), and root square mean errors of approximation (RSMEA < 0.05, Hooper et al., 2008).

Permutational multivariate analysis of variance (PERMANOVA) using distance matrices was carried out in the Vegan package (Oksanen et al., 2013) to evaluate the effects of compost addition, growth stage, and their interaction on AM fungal community composition. Subsequently, the AM fungal community composition was ordinated using non-metric multidimensional scaling (NMDS) with the dissimilarity matrices using the ‘metaMDS’ function in the Vegan package (Oksanen et al., 2013). Using the ‘envfit’ function of the Vegan package with 999 permutations, the treatments were fitted as centroids onto the ordination graphs, and the soil (pH, SM, SOM, TP, TN, AP, AN, AK, and MBC) variables were fitted as vectors onto the ordination graphics. Mantel tests were applied to explore correlations between AM fungal communities and soil variables in the ecodist package (Goslee and Urban, 2007). In order to determine AM fungal indicator species for treatments, we conducted indicator species analysis (species with *Indval* values > 0.3 and *P* < 0.05 are strong indicators) using the function ‘indval’ in the labdsv package (Roberts, 2010). We then investigated the variation in phylogenetic clustering among the different AM fungal communities by computing NRI. NRI was calculated using package picante (Kembel et al., 2010). The analyses above were carried out in R (v.3.1.1) (R Core Team, 2013).

## Results

### AM Root Colonization, ERH Density, and Spore Density

**FIGURE 1:**
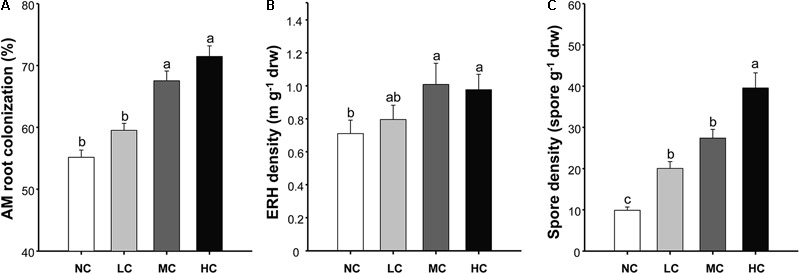
Arbuscular mycorrhizal (AM) root colonization **(A)**, extraradical hyphal (ERH) density **(B)**, and spore density **(C)** among treatments. NC, control; LC, low level of compost addition; MC, moderate level of compost addition; HC, high level of compost addition. Bars are SEMs (*n* = 12). Shared letters above bars denote no significant difference among treatments, as indicated by Tukey’s HSD test at *p* < 0.05.

The AM root colonization was significantly affected by both compost addition and growth stage, but AM fungal ERH density and spore density were only affected by compost addition (**Table 1**). However, no interactive effect between compost addition and growth stage was observed. Moderate and high levels of compost addition significantly increased AM root colonization and ERH density compared with control, whereas low level of compost addition did not cause significant increase in AM root colonization and ERH density (**Figures 1A,B**). Moreover, AM fungal spore density was significantly enhanced by all the compost rates compared with control (**Figure 1C**). The temporal variations analysis revealed that, AM root colonization in seedling stage was significantly lower than in flowering and mature stage (Supplementary Figure S1).

**Table 1:** Two-way ANOVAs examining the effects of compost addition (C), growth stage (G), and their interaction (C × G) on arbuscular mycorrhizal (AM) root colonization, spore density, extraradical hyphal (ERH) density, OTU richness, Shannon diversity index (*H*), and net-relatedness index (NRI).

	C		G		C × G	
Variables	*F*	*P*	*F*	*P*	*F*	*P*
Root Colonization	34.45	<0.001	6.51	0.004	1.15	0.36
Spore density	25.00	<0.001	0.23	0.80	0.19	0.98
ERH density	5.81	0.002	2.21	0.12	0.45	0.84
OTU richness	0.2	0.90	1.58	0.22	0.26	0.95
*H*	0.63	0.60	1.27	0.29	0.38	0.88
NRI	0.71	0.55	4.76	0.02	0.39	0.88

**FIGURE 2:**
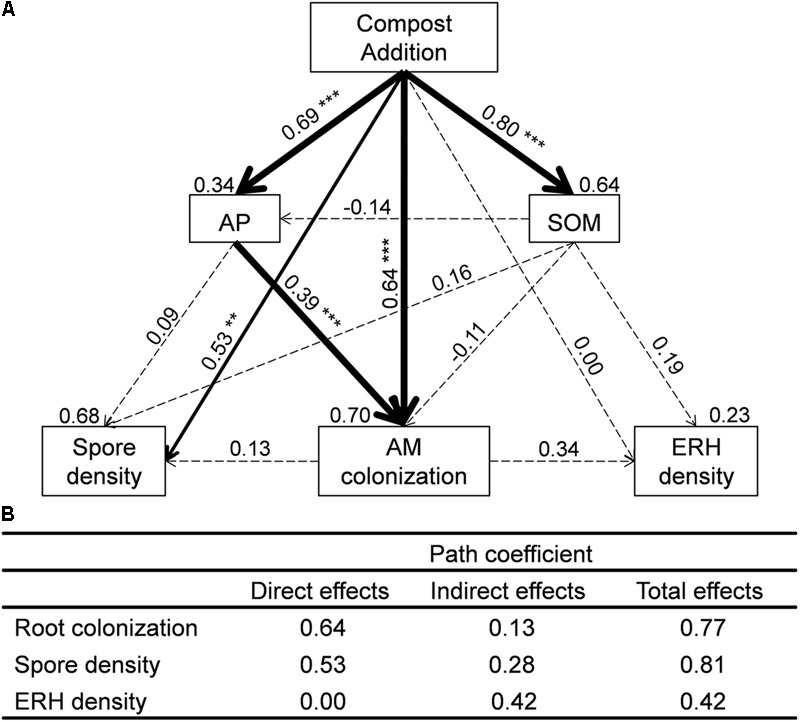
**(A)** Structural equation models (SEMs) showing the direct and indirect effects of compost addition on arbuscular mycorrhizal (AM) colonization, spore density, and extraradical hyphal (ERH) density. Values above the line represent the path coefficients. Values above the text box represent the residuals. Solid and dashed lines indicate significant and non-significant pathways, respectively. Width of arrows indicates strength of the standardized path coefficient (^∗∗∗^*P* < 0.001; ^∗∗^*P* < 0.01; ^∗^*P* < 0.05). **(B)** Impact of compost addition on AM root colonization, spore density, and ERH density assessed by SEM including direct, indirect, and total effect coefficients based on hypothesized causal relationships. SOM, soil organic matter; AP, available phosphorus.

We used SEM to assess direct and indirect effects of compost addition on AM root colonization, spore density, and ERH density (**Figure 2**). The final SEM models met our significance criteria (χ^2^ = 1.28, *df* = 2, *P* = 0.53, RMSEA < 0.001, GFI = 0.99, AIC = 39.28) and were able to explain 70% of AM root colonization, 68% of AM fungal spore density, and 23% of AM fungal ERH density (**Figure 2**). AM root colonization was significantly influenced by compost addition directly (λ = 0.64, **Figure 2**) and indirectly (mediated through soil AP, λ = 0.13, **Figure 2**). AM fungal spore density was significantly influenced by compost addition directly (λ = 0.53, **Figure 2**), while AM fungal ERH density was influenced by compost addition indirectly (λ = 0.42, **Figure 2**).

### MiSeq Sequencing Analysis and Identification of AM Fungi

A total of 2,355,017 reads were obtained after a quality control procedure, from which 68,892 potential chimeras were removed. The remaining 2,286,125 non-chimeric reads were assigned to 186 OTUs based on a 97% sequence similarity. Of these 186 OTUs, 48 (1,984,995 reads) belonged to AM fungi. As the number of AM fungal reads ranged from 12,516 to 68,300 among the samples, the read numbers were normalized to 12,516, resulting in a normalized dataset containing 45 AM fungal OTUs (600,004 reads).

**FIGURE 3:**
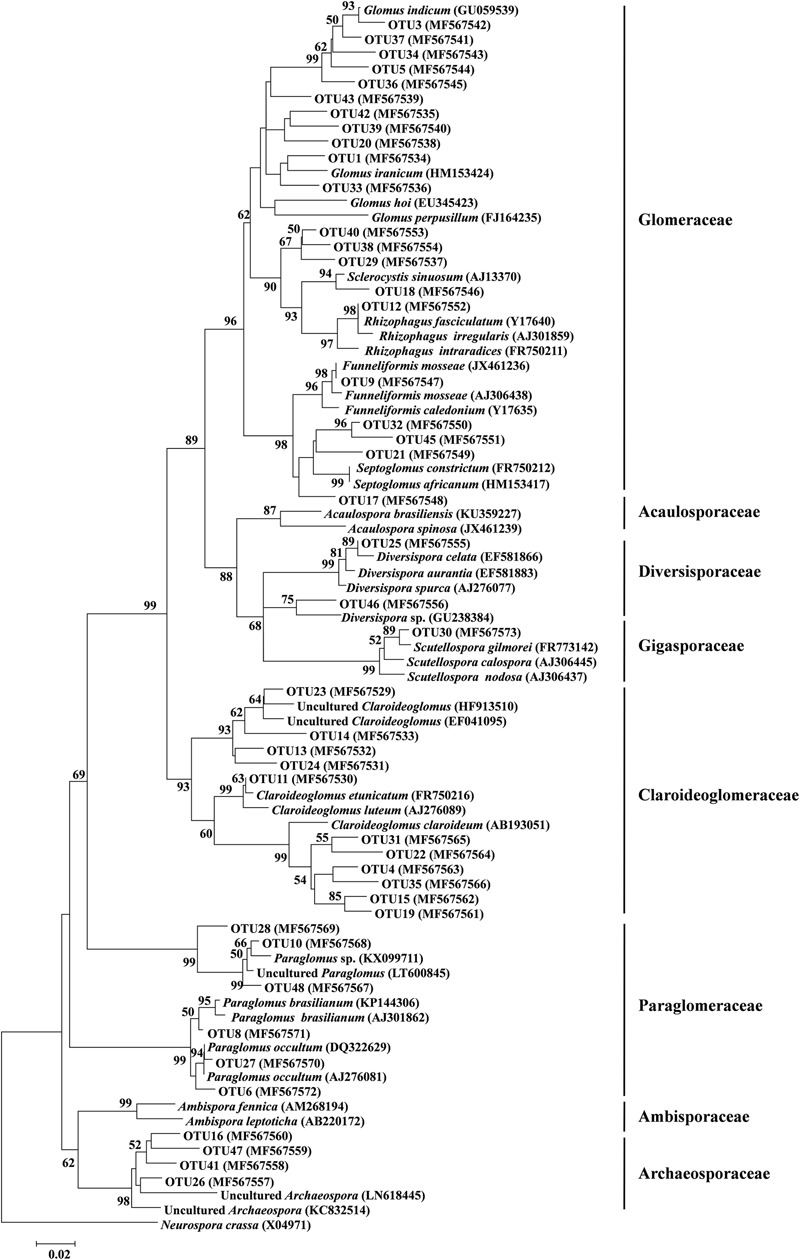
Neighbor-joining tree of arbuscular mycorrhizal (AM) fungi. The 18S rDNA sequences (ca. 334 bp) of AM fungal OTUs obtained in this study and the references downloaded from GenBank were shown concomitantly by the corresponding accession numbers (parentheses). Bootstrap values were calculated on the basis of 1,000 data resampling (> 50% of the values are shown). *Neurospora crassa* was used as an outgroup. Scale bar represents 2% sequence divergence.

Of these 45 AM fungal OTUs, the first 11 frequent OTUs occurred in ≥ 24 (50%) soil samples, and the 16 least frequent OTUs occurred in ≤ 5 (10.4%) soil samples (Supplementary Figure S2A). The first 10 abundant OTUs (each > 20,000 reads) accounted for 81.8% of the total AM fungal reads, and the remaining 35 OTUs accounted for 18.2% (Supplementary Figure S2B). Among these 45 AM fungal OTUs, 21 belonged to Glomeraceae (11 *Glomus*, 3 *Septoglomus*, 1 *Rhizophagus*, 1 *Sclerocystis*, 1 *Funneliformis*, 4 unidentified genera), 11 to Claroideoglomeraceae (11 *Claroideoglomus*), 6 to Paraglomeraceae (6 *paraglomus*), 4 to Archaeosporaceae (4 unidentified genera), 2 belong to Diversisporaceae (2 *Diversispor*a), and 1 to Gigasporaceae (1 *Scutellospora*, **Figure 3**). A rarefaction analysis showed that all rarefaction curves for observed AM fungal OTUs reach the saturation platform, indicating that sequencing effort was sufficient to identify the most AM fungi in this study (Supplementary Figure S3).

### AM Fungal Community

**FIGURE 4:**
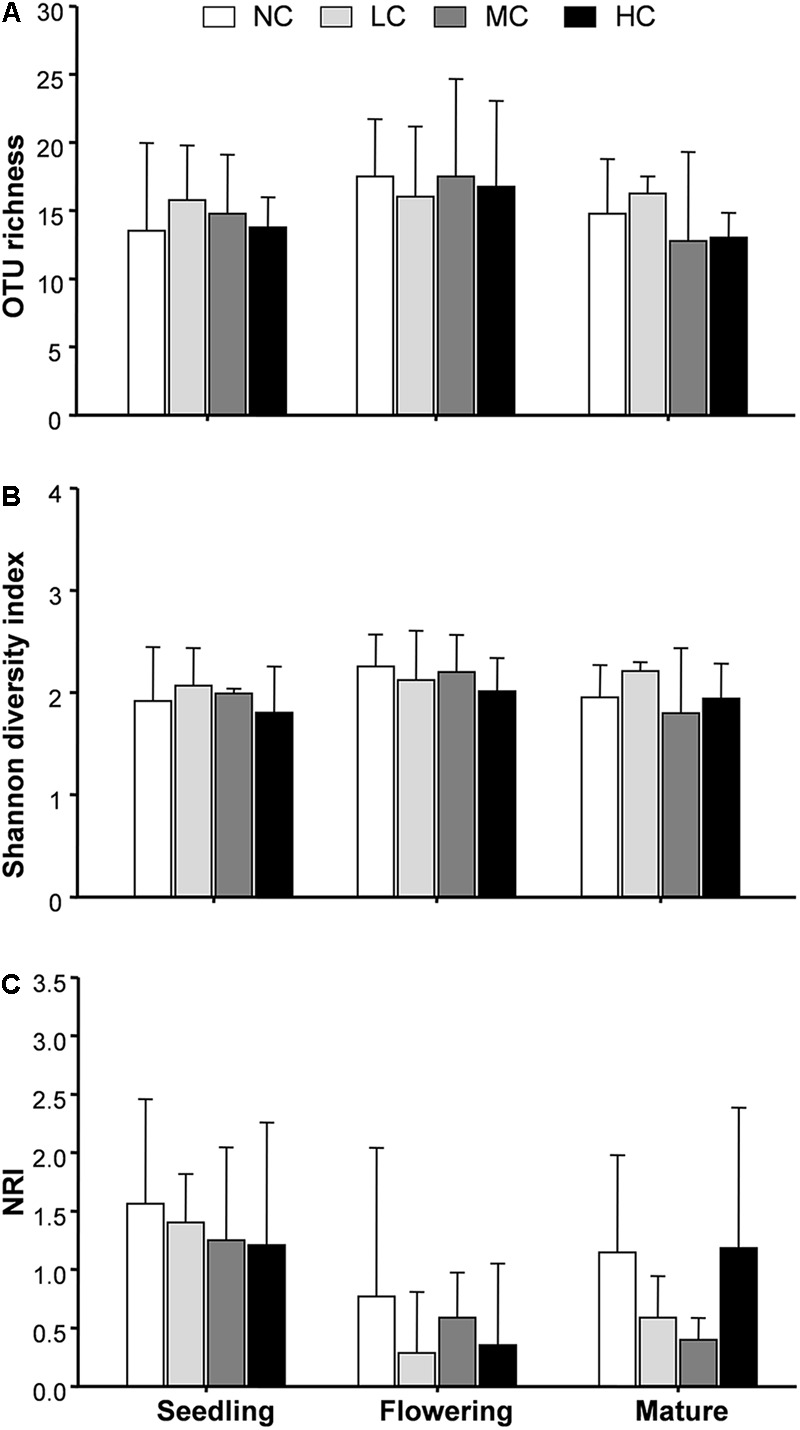
Arbuscular mycorrhizal fungal operational taxonomic unit (OTU) richness **(A)**, Shannon diversity index **(B)**, and net-relatedness index (NRI, **C**) among treatments. NC, control; LC, low compost addition; MC, moderate compost addition; HC, high compost addition. Bars are SDs of the means (*n* = 4).

**FIGURE 5:**
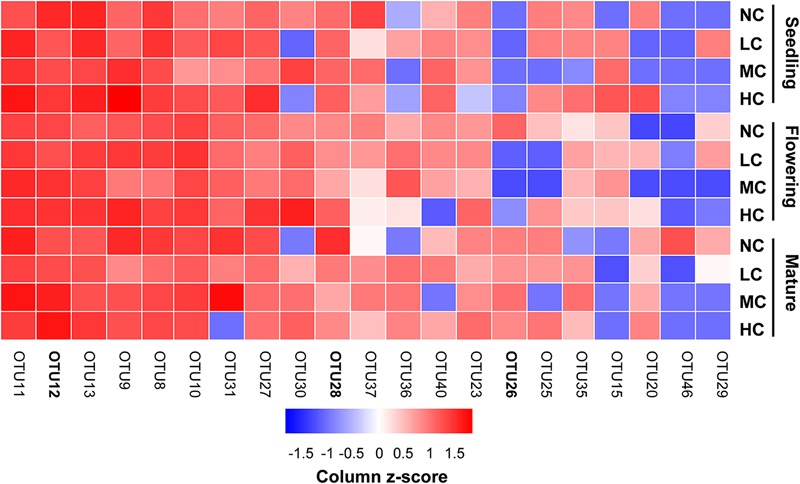
Relative abundances [log_2_ (*x*+1)] of the most prevalent combined ‘top 10’ OTUs observed in seedling, flowering, and mature stage in soil. Color gradients indicate taxa relative abundances, with red colors indicating prevalent taxa and blue colors indicating rare taxa in soil. The OTUs in bold indicate indicator OTUs. NC, control; LC, low level of compost addition; MC, moderate level of compost addition; HC, high level of compost addition.

Two-way ANOVA analysis showed that compost addition, growth stage, and their interaction showed no significant effect on AM fungal OTU richness and Shannon diversity indices (**Figures 4A,B** and **Table 1**). Indicator species analyses showed that OTU 26 (*Archaeospora* sp.) was indicator in NC treatment in flowering stage (*Indval* value = 0.75, *P* = 0.03), which did not occur in low, moderate, or high compost addition treatments (**Figure 5**). In mature stage, OTU 12 (*R*. *fasciculatum*) was indicator in HC treatment (*Indval* value = 0.47, *P* = 0.04), while OTU 28 (*Paraglomus* sp.) was indicator in NC treatment (*Indval* value = 0.68, *P* = 0.04). Moreover, the abundance of *R*. *fasciculatum* was generally increased along with the compost addition gradient (**Figure 5**), while *Paraglomus* sp. showed an opposite trend (**Figure 5**). However, no indicator species was observed in seedling stage.

**FIGURE 6:**
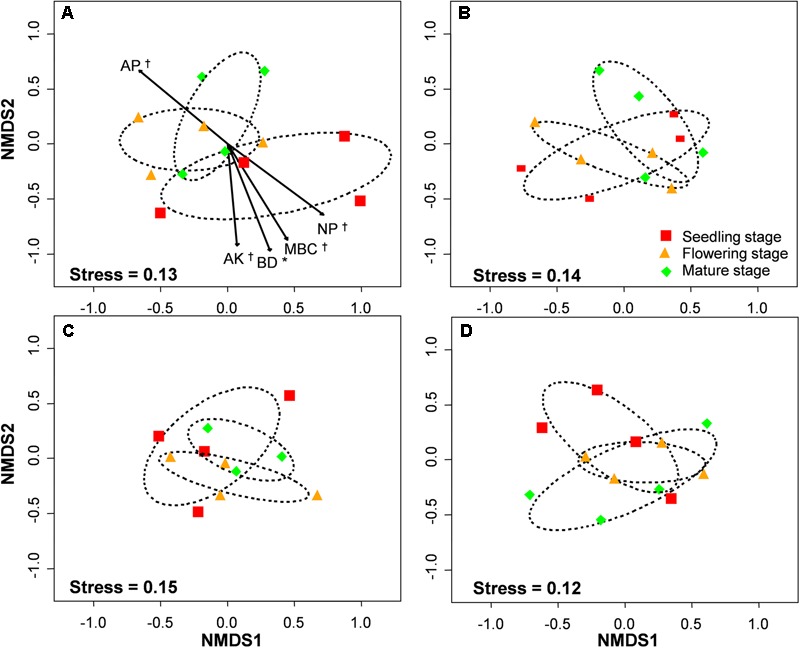
Non-metric multidimensional scaling (NMDS) of arbuscular mycorrhizal fungal community composition in control **(A)**, low level of compost **(B)**, moderate level of compost **(C)**, and high level of compost **(D)** addition treatment. Circles with dashed line in NMDS plot are 95% confidence ellipses of seedling, flowering, and mature stages. AP, available P; AK, available K; BD, bulk density; MBC, microbial biomass carbon; N:P, N:P ratio. ^∗^*P* < 0.05; ^†^0.05 < *P* < 0.1.

The PERMANOVA analysis showed that the AM fungal community composition was unaffected by compost addition (*r*^2^ = 0.02, *P* = 0.34), but marginally affected by growth stage (*r*^2^ = 0.04, *P* = 0.05), and significantly affected by their interaction (*r*^2^ = 0.06, *P* = 0.001). Mantel test showed that AM fungal community composition was significantly related to growth stage, N:P ratio and microbial community composition, and marginally affected by MBC (**Table 2**). By an analysis of interactive effect between compost addition and growth stage, AM fungal community composition was marginally affected by growth stage in NC treatment (*r*^2^ = 0.46, *P* = 0.06, **Figure 6A**), but not in LC (*r*^2^ = 0.21, *P* = 0.36, **Figure 6B**), MC (*r*^2^ = 0.07, *P* = 0.75, **Figure 6C**), and HC (*r*^2^ = 0.24, *P* = 0.31) treatment (**Figure 6D**) through NMDS analysis. Furthermore, AM fungal community composition was significantly related to bulk density, and marginally related to MBC, AP, AK, and N:P ratio in NC treatment (**Figure 6A**). However, AM fungal community composition was unaffected by any soil variable in LC, MC, and HC treatment (**Figures 6B–D**).

**Table 2:** Mantel tests of the arbuscular mycorrhizal community with growth stage, compost addition, pH, soil organic matter (SOM), available N (AN), available P (AP), available K (AK), N:P ratio, soil microbial biomass carbon (MBC), and community.

	*r*	*P*
Growth stage	0.09	0.01
Compost addition	0.01	0.36
pH	0.00	0.51
SOM	-0.04	0.71
AN	0.00	0.52
AP	0.03	0.33
AK	0.02	0.36
N:P ratio	0.15	0.04
MBC	0.11	0.08
Microbial community	0.21	0.02

We next used NRI to explore AM phylogenetic relationship. NRI values were significantly (*P* < 0.05) different from zero in all growth stages. Two-way ANOVA analysis indicated that the NRI was unaffected by compost addition, but significantly affected by growth stage. Moreover, NRI was significantly higher in seedling stage than flowering stage (**Figure 4C**).

## Discussion

Our results indicated that compost addition enhanced AM root colonization, ERH density, and spore density. These results were consistent with previous studies that compost addition most often had positive effect on AM growth and sporulation (Labidi et al., 2007; Tanwar et al., 2013; Cavagnaro, 2015). The positive effect of compost on AM fungi is possibly due to several reasons. First, compost is rich in humic acid, which were reported to stimulate AM hyphal growth and sporulation (Gryndler et al., 2009). Second, SEM analysis indicated that compost addition enhanced AM root colonization likely through soil AP. It was reported that legume crops (e.g., soybean) have relatively high phosphorus demand due to the formation of root nodules (Scheublin et al., 2004). Consequently, if soybean is P limited and AM fungi may not, it will allocate carbon to AM (Treseder and Allen, 2002). In present study, compost provide a sustained release of P rather than a large single pulse of P, and maintained moderate level of soil AP (Yang et al., 2017). Therefore, AM fungi will proliferate when the soil AP concentration was sufficient for AM growth (Treseder and Allen, 2002) but not for the crop. Third, it was reported that the N concentration in the external hyphae of AM fungi is 4–7 times greater than that of plant shoots and at least 10 times greater than that of roots (Hodge and Fitter, 2010). Consequently, AM fungi must have a high N demand and require large amounts of N for their own growth (Hodge et al., 2010), and this can explain why AM fungal growth and sporulation were stimulated by N-rich compost. Although the addition of compost could introduce a myriad of microbes, however, Saison et al. (2006) showed that a microbial community from compost will be outcompeted by the native microbial community once the compost is applied to the soil. In addition, the compost we used was obtained from cow manure and maize straw which did not contain soil or roots, and no AM fungal spore was detected in the compost. Therefore, the effect of compost addition on AM was not mediated by the bacteria or AM fungal propagules in the compost.

Pearson’s correlation analysis indicated a positive and dose-dependent effect of compost addition on AM root colonization, ERH density, and spore density existed (Supplementary Table S1). These results were in accordance with several studies (Valarini et al., 2009; Tanwar et al., 2013). For instance, Valarini et al. (2009) reported that AM root colonization significantly increased along with compost addition gradient (0, 8, 20, and 30 Mg/ha) in wheat and bean roots. However, in a pot experiment reported by Cavagnaro (2014), AM colonization in *Solanum lycopersicum* roots showed a parabolic trend and peaked in 12.5 Mg/ha municipal greenwaste compost addition treatment. Although Cavagnaro (2014) did not report the soil AP level during the growing season, the high level of available P in the compost (available P: 1459 ± 30 mg/kg, much higher than in this study) could have maintained a high level of AP. Moreover, the C:N ratio was 11.4 ± 0.2 in Cavagnaro (2014), which was quite lower than the compost used in our study (C:N ratio: 21.0). The low C:N ratio allows mineralization by soil microorganism and release large amount of P into soil. As a consequence, the excessive P will cause reduction in AM root colonization. In addition, Copetta et al. (2011) observed a decreasing trend of AM root colonization in *S. lycopersicum* along with compost addition gradient (compost concentration of 0, 25, 50, 75, and 100%) in pot experiment. These inconsistent results indicated that AM fungi may show various responses to different rates of compost addition depending on the compost type (Copetta et al., 2011; Cavagnaro, 2014), plant species (Copetta et al., 2011; Cavagnaro, 2014), and dosage of compost (Copetta et al., 2011). Anyhow, a rate of 45 Mg/ha compost did not inhibit AM growth and was most effective for AM growth in present study.

In this study, AM root colonization was subjected to obvious temporal variations and significantly lower in seedling stage than flowering and mature stage. The combined effect of soil nutrient availability and plant carbon allocation could be responsible for this result (Dumbrell et al., 2011; Bainard et al., 2012). However, the AM root colonization in seedling stage was a little high, as the plants were just 1 month old. The high AM root colonization in seedling stage may be attributed to several reasons. First, Hart and Reader (2002) reported Glomaceae isolates were generally the fastest colonizers of plant roots. Moreover, in a pot experiment reported by Vangelisti et al. (2018), AM colonization (*Rhizoglomus irregular*, Glomeraceae) of sunflower was 7.3 ± 1.3% after four days. Interestingly, we found Glomeraceae was the most abundant group in soil in present study. The second reason may be ascribed to the drought stress. The precipitation was extremely low during the seedling stage (37 mm in May, 2016) at our study site. It was reported that soil AP becomes less available under drought stress. Thus, AM colonization can be extremely important for nutrient uptake (Saia et al., 2014a) and thereby increase AM root colonization even at earlier stage of growth. In addition, we have a low density (30 plants per square meter) and fast growing species, with sturdy N fixing ability and thick root system. This condition can probably force the plant to rapidly activate the AM symbiosis to take up inorganic nutrients.

Predominance of Glomeraceae in AM fungal communities was in agreement with previous reports for agroecosystem (Oehl et al., 2004; Beauregard et al., 2013; Njeru et al., 2015). Among the abundant AM fungal species, *Rhizophagus fasciculatum* showed increasing trend along with compost addition gradient, whereas the opposite was observed with *Paraglomus* sp. Similarly, Yu et al. (2013) examined the effect of organic fertilizer (Protamylasse) on AM fungal community through 454 pyrosequencing in *Pisum sativum* roots, and reported that *Paraglomus sp.* was more abundant in treatments with low level than high level of organic fertilizer treatment. Whereas most AM fungal OTUs detected in our study remained stable to compost addition. This contrasting response of different AM fungi to compost addition indicated different life strategies of AM fungi (Hart and Reader, 2002). Given that AM fungi differ substantially in their ability to acquire and supply nutrients to plants (Koch et al., 2004; Munkvold et al., 2004), they may also be differently affected by compost mineralization in the soil.

Given that compost addition enhanced plant growth (Lee et al., 2004) and altered carbon allocation (Donn et al., 2014) to plant roots, AM may be affected by compost addition. However, we did not detect the compost effect on AM fungal diversity and community in soil. Although very limited data was available, the effect of other organic fertilizer could be referenced. For instance, 1-year organic fertilization did not significantly affect AM fungal OTU richness in onion roots (Galván et al., 2009). It was also found that 2-year organic manure addition did not significantly increase AM fungal OTU richness and modify community composition in maize (Toljander et al., 2008; Beauregard et al., 2013) and *Avena sativa* (Zheng et al., 2016) rhizosphere soil. Therefore, AM fungal community composition in short-time C addition experiments could be strongly affected by the plant species and scarcely by the compost itself.

The NRI analysis and Mantel test revealed a significant temporal variation in AM fungal community during growing season, which is supported by a large number of previous studies (Dumbrell et al., 2011; Wu et al., 2011; Bainard et al., 2012; Barnes et al., 2016). It has been suggested that the temporal variation in AM fungal community composition is likely a response to plant and fungal phenology and the soil variables (Bever et al., 2001; Dumbrell et al., 2011). For instance, both soil N and P availability play key roles in shaping AM fungal community composition (Liu et al., 2012), but N and P have different effects on AM depending on soil P or N limitation (Johnson et al., 2015). Therefore, available N:P ratio could actually be a more important driver than just P or N levels independently in shaping AM fungal community composition (Roy et al., 2017). In present study, N:P ratio was significantly different among three growth stages and significantly affected AM fungal community composition as confirmed by Mantel test. Therefore, N:P ratio could be one reason for AM fungal community dynamics. Moreover, soil microbial community composition was significantly different among three growth stages (Yang et al., 2017) at the same study site. It was reported that some bacteria can affect AM fungal germination and growth (Carpenter-Boggs et al., 1995); other bacteria can influence the physiology of the plants (Saia et al., 2015a,b). The soil bacteria may thus directly and indirectly affect AM fungal community composition, which was identified by Mantel test. We further found that the temporal variation in AM fungal community only occurred in control treatment, but not in low, moderate, and high compost addition treatments in present study. In the same way, Wang et al. (2015) failed to reveal community dynamics of AM fungi in high-input rice cultivation systems through clone library analysis. In compost addition treatment, AM will receive adequate carbon from host and their growth would not be regulated by dynamics of carbon allocation during the growing season (Dumbrell et al., 2011). In addition, AM fungal community in control treatment was shaped by several soil variables (e.g., bulk density, AK, and AP) in present study, but no significant soil variables was observed in compost addition treatments. Therefore, a relatively high nutrient level maintained by compost in soil may remove the nutrient limitation for AM and thus remove the temporal change in AM fungal community composition in compost addition treatment.

In addition, we determined the AM fungal community composition in soil but not in roots. It was reported that there can be conspicuous difference in AM fungal community composition between soil and roots due to the different ecological and evolutionary forces (Liu et al., 2012; Yang et al., 2013). Previous studies have shown that AM fungal community showed different response to environmental variations (Yang et al., 2013; Liu et al., 2016) between soil and roots. Therefore, it is likely that compost addition impacted AM fungal community composition in roots rather than soil in present study.

## Conclusion

The response of AM fungal community to combined compost addition and growth stage was investigated in soybean agroecosystem on the Songnen Plain. Our results indicated that 1-year compost addition significantly enhanced the AM root colonization, ERH density, and spore density. However, AM fungal OTU richness and community composition were unaffected by compost addition. Furthermore, the temporal variation in AM fungal community only occurred in control treatment, but not in low, moderate, and high compost addition treatments. Our findings indicated that growth stage is a stronger determinant than 1-year compost addition in shaping AM fungal community in black soil of Northeast China.

## Author Contributions

WY, SG, WS, AB, and XX planned and designed the research. WY, YX, and WS carried out the research and conducted the fieldwork. WY, SG, AB, and XX wrote the manuscript.

## Conflict of Interest Statement

The authors declare that the research was conducted in the absence of any commercial or financial relationships that could be construed as a potential conflict of interest.
