# Supplementary material for: Compost Addition Enhanced Hyphal Growth and Sporulation of Arbuscular Mycorrhizal Fungi without Affecting Their Community Composition in the Soil
Source: Front Microbiol. 2018 Feb 7;9:169. doi: 10.3389/fmicb.2018.00169 (PMC5808307; doi:10.3389/fmicb.2018.00169)
Supplement: Supplementary file 1 [file Data_Sheet_1.pdf]

Compost Addition Enhanced Hyphal Growth and Sporulation of Arbuscular Mycorrhizal Fungi without Affecting Their Community Composition in the Soil

Wei Yang[1], Siyu Gu[1], Yuting Guo[1], Ying Xin[1], Xiuhong Xu[1*]

1. College of Resources and Environment, Northeast Agricultural University, Harbin 150030, China

* Corresponding author

Email: xuxiuhong@neau.edu.cn

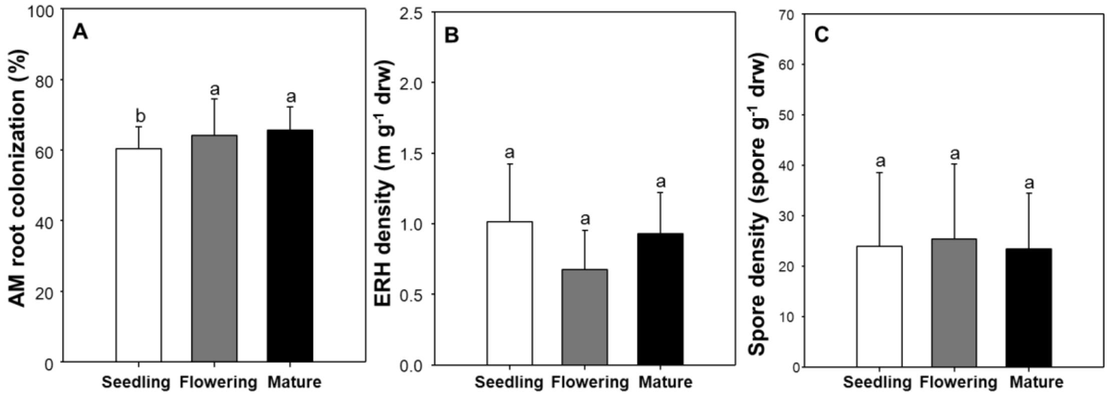

Figure. S1 Arbuscular mycorrhizal (AM) root colonization (A), extraradical hyphal (ERH) density (B) and spore density (C) among growth stages. Bars are standard errors (n = 16). Shared letters above bars denote no significant difference among treatments and growth stages respectively, as indicated by Tukey's HSD test at $P < 0.05$.

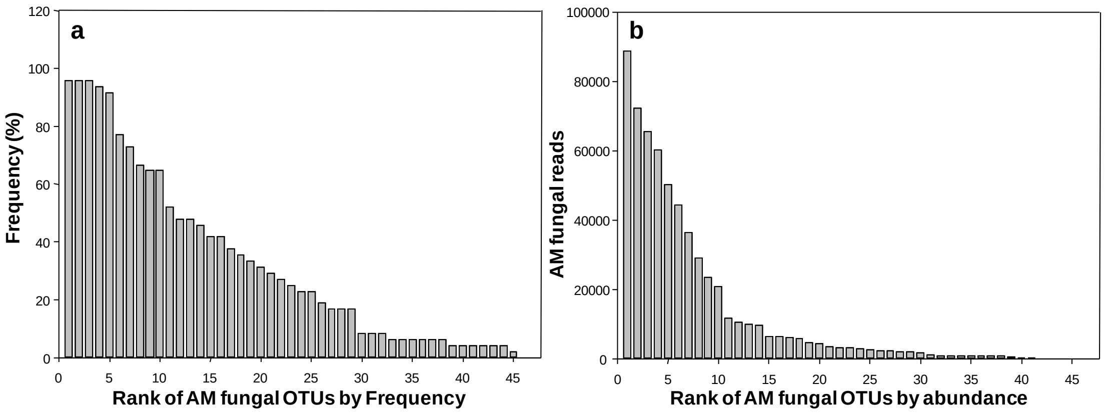

Figure. S2 Rank of AM fungal operational taxonomic units (OTUs) by frequency (a), and rank of AM fungal OTUs by abundance (b).

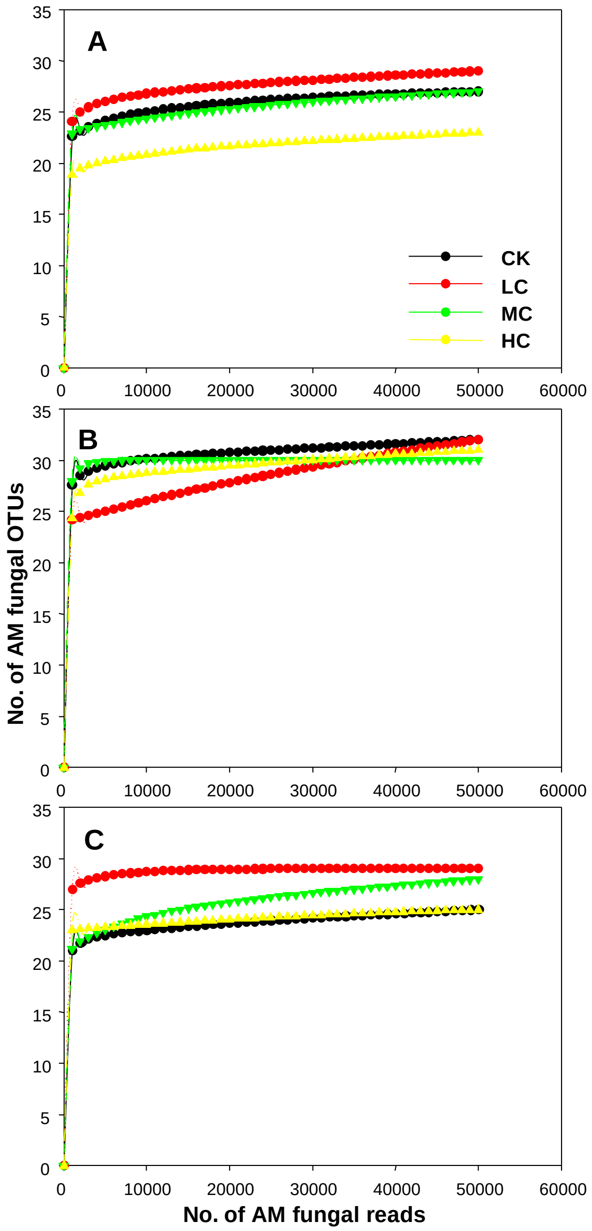

Figure. S3 Rarefaction curves for the observed arbuscular mycorrhizal (AM) fungal OTUs among treatments in seedling (a), flowering (b) and mature stage (c). Abbreviations: NC, control; LC, low level of compost addition; MC, moderate level of compost addition; HC, high level of compost addition.

Table S1 Pearson correlation between AM fungal variables and soil variables.

		time	compost cocentration	spore density	CR	ERH	pH	SOM	AN	AP	TP	AK	TN	MBC	C: N	N: P
time	r	1														
compost concentration	r	0.000	1													
spore density	r	-.018	.812**	1												
CR	r	.278	.787**	.690**	1											
ERH density	r	-.004	.447**	.360*	.454**	1										
pH	r	.288*	.328*	.347*	.514**	.273	1									
SOM	r	.151	.791**	.690**	.557**	.386**	.146	1								
AN	r	.039	.362*	.266	.391**	.144	.311*	.210	1							
AP	r	.404**	.564**	.540**	.708**	.270	.696**	.360*	.498**	1						
TP	r	-.442**	.521**	.446**	.320*	.108	.276	.258	.459**	.460**	1					
AK	r	-.366*	.527**	.483**	.429**	.148	.398**	.166	.387**	.487**	.663**	1				
TN	r	.428**	.413**	.299*	.484**	.080	.486**	.338*	.386**	.644**	.380**	.274	1			
MBC	r	-.527**	.125	.073	-.096	.127	-.371**	.118	-.020	-.364*	.111	.132	-.278	1		
C: N	r	-.052	.655**	.625**	.380**	.357*	-.057	.901**	.024	.096	.107	.076	-.082	.222	1	
N: P	r	-.527**	-.488**	-.472**	-.584**	-.145	-.489**	-.386**	-.207	-.746**	-.185	-.320*	-.601**	.438**	-.159	1

Abbreviations: CR, colonization rate; ERH, extraradical hyphae; SOM, soil organic matter; AN, available N; AP, available P; TP, total P; AK, available K; TN, total N; MBC, microbial biomass carbon
